# Personal

**Published:** 1898-03

**Authors:** 


### Personal.

Dr. Benjamin Lee, of Philadelphia, has recently been appointed health officer of that city. He is a son of the late Bishop Lee, of Delaware. During the war he acted as surgeon in the 22d Regiment N. Y. N. G. He has been a close student of hygiene, and is a member of many learned societies. As secretary of the state board of health of Pennsylvania for a number of years, he rendered important service to the cause of sanitation, converting the organisation from a mere bureau of vital statistics into an active and efficient agency for promoting the health of the people.

Dr. James F. W. Ross, of Toronto, read a paper before the Buffalo Academy of Medicine, Tuesday evening, January 25, 1898, entitled Torn and punctured wounds of the uterus and vagina; symptoms, prognosis and treatment.

